# Randomly Methylated *β*-Cyclodextrin Inclusion Complex with Ketoconazole: Preparation, Characterization, and Improvement of Pharmacological Profiles

**DOI:** 10.3390/molecules29091915

**Published:** 2024-04-23

**Authors:** Yili Ding, Shufeng Xu, Charles Ding, Zhiyuan Zhang, Zhe Xu

**Affiliations:** 1 College of Science, Mathematics and Technology, Wenzhou-Kean University, Wenzhou 325060, China; xzhe@kean.edu; 2 Dorothy and George Hennings College of Science, Mathematics and Technology, Kean University, 1000 Morris Ave, Union, NJ 07083, USA; 3 Life Science Department, Foshan University, Foshan 528000, China; 4 Keck School of Medicine, University of Southern California, Los Angeles, CA 90089, USA; dingchar@usc.edu

**Keywords:** ketoconazole, randomly methylated *β*-cyclodextrin, inclusion complex, water solubility, in vitro and in vivo pharmacokinetic study

## Abstract

As a powerful imidazole antifungal drug, ketoconazole’s low solubility (0.017 mg/mL), together with its odor and irritation, limited its clinical applications. The inclusion complex of ketoconazole with randomly methylated *β*-cyclodextrin was prepared by using an aqueous solution method after cyclodextrin selection through phase solubility studies, complexation methods, and condition selection through single factor and orthogonal strategies. The complex was confirmed by FTIR (Fourier-transform infrared spectroscopy), DSC (differential scanning calorimetry), TGA (thermogravimetric analysis), SEM (scanning electron microscope images), and NMR (Nuclear magnetic resonance) studies. Through complexation, the water solubility of ketoconazole in the complex was increased 17,000 times compared with that of ketoconazole alone, which is the best result so far for the ketoconazole water solubility study. In in vitro pharmacokinetic studies, ketoconazole in the complex can be 100% released in 75 min, and in in vivo pharmacokinetic studies in dogs, through the complexation, the *C*_max_ was increased from 7.56 μg/mL to 13.58 µg/mL, and the AUC_0~72_ was increased from 22.69 μgh/mL to 50.19 μgh/mL, indicating that this ketoconazole complex can be used as a more efficient potential new anti-fungal drug.

## 1. Introduction

**Figure 1:**
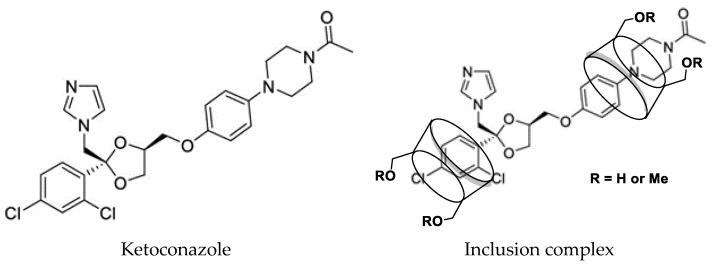
Structures of ketoconazole and its inclusion complex with randomly methylated *β*-cyclodextrin.

As an imidazole antifungal drug (Figure 1), ketoconazole was used to inhibit the production of fungal cell membranes and treat a diversity of deep and superficial tissue fungal infections including dermatophytes, chronic mucocutaneous candidiasis, fungal infections, of the gastro-intestinal tract, fingernails not responding to topical treatment, and systemic infections such as blastomycosis, candidiasis, coccidioidomycosis, histoplasmosis, paracoccidioidomycosis, and oropharyngeal candidiasis [1]. Ketoconazole as a pH-dependent binary compound (pKa = 2.94, 6.51) is very soluble in conditions with pH below 3 (e.g., in a typical acidic stomach) but precipitates at higher pH levels (e.g., in the small intestine) with low water solubility (0.017 mg/mL) [2]. It is classified as a class II drug in the biopharmaceutics classification scheme due to its high permeability and insufficient solubility in gastrointestinal fluids under normal conditions [3].

It is common for compounds with aqueous solubilities lower than 0.1 mg/mL to have a dissolution limitation of absorption, which is one of the causes of local irritation to the gastric mucosa leading to gastrointestinal side effects [4]. The insolubility of ketoconazole in water, its instability, odor, and irritation greatly limit its clinical applications [5].

Cyclodextrins, as excellent drug carriers, were used to increase ketoconazole’s water solubility for many years. For example, *β*-cyclodextrin was used to form the inclusion complex with ketoconazole a in 1:2 molar ratio by freeze-drying, solvent evaporation, and ultrasonic methods to enhance the nasal absorption and the native fluorescence of the drug. The aqueous solubility was increased from 0.017 mg/mL to 2.58 mg/mL [6,7,8,9,10,11] through the complexations of ketoconazole with *β*-cyclodextrin and citric acid, with *β*-cyclodextrin, and with HP-*β*-cyclodextrin [12,13,14]. Tartaric acid could form the non-covalent bond with a ketoconazole complex, as confirmed by the mass spectrum [15]. A solubility diagram of type A_L_ was found when ketoconazole was complexed with HP-*β*-cyclodextrin, while the A_P_ type was found when ketoconazole was complexed with randomly methylated *β*-cyclodextrin in the presence of water-soluble polymers such as polyvinylpyrrolidone, hydroxypropyl-methylcellulose, and sodium carboxymethyl-cellulose [16]. Different hydroxy acids, such as citric acid, tartaric acid, and malic acid, were used to increase the aqueous solubility of ketoconazole [11,12].

*β*-Cyclodextrin, *γ*- and dimethyl *β*-cyclodextrins could enhance the solubility and dissolution rate of ketoconazole [17]. The complex of ketoconazole with HP-*β*-cyclodextrin and randomly methylated *β*-cyclodextrin by using physical mixing, kneading, and spray-drying methods increased the water solubility of ketoconazole by 111-fold [16,18,19,20].

Based on the assumption of un-complexed guest percentages semi-quantitatively by DSC curves for the complexes with *β*- and *γ*-cyclodextrins, heptakis (2,6-di-*O*-methyl)-*β*-cyclodextrin, heptakis (2,3,6-tri-*O*-methyl)-*β*-cyclodextrin, HP-*β*-cyclodextrin and carboxymethyl-*β*-cyclodextrin, it was found that the complexes from ketoconazole and HP-*β*-cyclodextrin (1:1 and 1:2) gave the best solubility increasing results [21], and the interactions between HP-*β*-cyclodextrin and ketoconazole in the ternary system were elucidated through microscale thermophoresis and NMR spectroscopy study [22], and the results indicated that the ternary system of microemulsion combination with HP-*β*-cyclodextrin may be a promising approach for skin targeting delivery of ketoconazole.

The formation of the inclusion complexes between ketoconazole and cyclodextrins is markedly affected by the pH value of the solutions [23]. When ketoconazole formed the inclusion complexes with *β*-cyclodextrin in 1:1 and 1:2 ratios, the solubility of the ketoconazole in 0.1 N HCl was increased from 0.096 to 17.9 mg/mL and 19.8 mg/mL, respectively, and ketoconazole was released completely in 30 min [24].

The multicomponent complexes of ketoconazole with *β*-cyclodextrin and proline or *N*-acetylcysteine could enhance the solubility and antifungal activity of ketoconazole; however, due to the low content of the complex, the signals from ketoconazole in the complex could not been seen clearly in the NMR spectrum in D_2_O [25,26]. Moreover, when the complex of ketoconazole/*β*-cyclodextrin was prepared and loaded on cotton, the complex on the fabric showed good antifungal activity and slower release compared with the pure ketoconazole [27]. Silver nanoparticles were added during the formation of the complex between ketoconazole and *β*-cyclodextrin, and the resulting materials showed better antifungal and antibacterial activity than that of the drug alone; however, the proton NMR spectrum of the complex indicated that the complex only contained a trace amount of ketoconazole [28].

From the discussion above, it is obvious that even though great efforts were made in preparing the inclusion complex of ketoconazole with cyclodextrins, the water solubility of ketoconazole was still not significantly improved. In continuation of drug inclusion complex studies [29,30,31,32,33], there is further interest in improving the water solubility of ketoconazole through the formation of inclusion complexes with cyclodextrins. Through the complexation of ketoconazole with randomly methylated *β*-cyclodextrin after cyclodextrin selection using phase solubility study, complexation method, and condition selection using single factor and orthogonal strategies, the water solubility of ketoconazole was increased 17,000 times compared with that of the original drug, which was so far the best result for ketoconazole solubility study. The in vitro and in vivo pharmacokinetic profiles of the inclusion complex were evaluated. Based on these results, this ketoconazole inclusion complex can be used as a potential potent antifungal drug, and in this paper, we would like to report these results.

## 2. Results and Discussion

Through study of the UV spectra of ketoconazole, its maximum UV absorption wavelength of 254 nm was determined for HPLC analysis. From the HPLC analysis of a series of solutions of ketoconazole in methanol with different concentrations in the range of 0.06–0.21 mg/mL, the linear regression equation Y = 5.5505X + 0.2147 (coefficient of determination: R^2^ = 0.9973, Y: absorption peak area, X: concentrations) was obtained, and the HPLC standard curve was shown as follow.


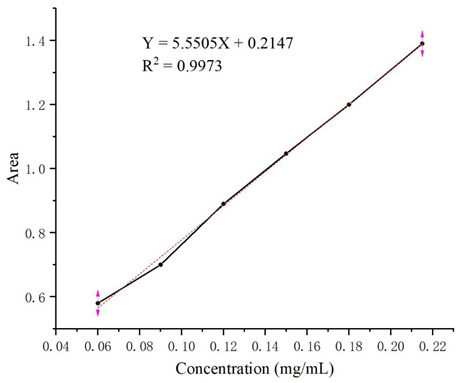


**Figure 2:**
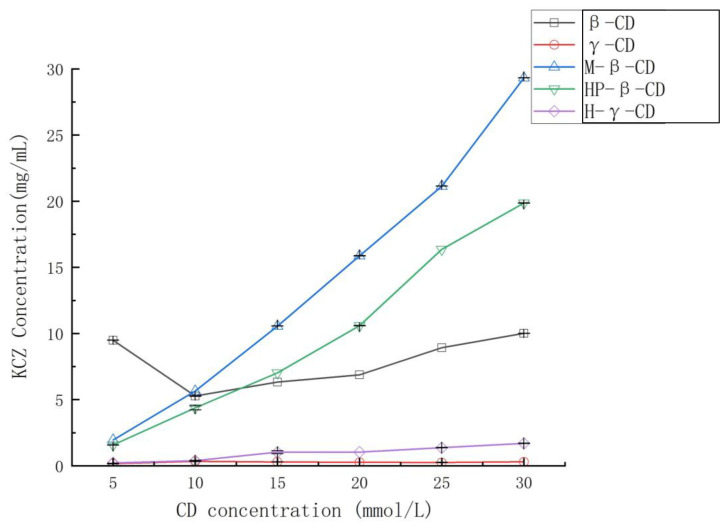
Phase solubility curves of ketoconazole with *β*-cyclodextrin, *γ*-cyclodextrin, randomly methylated *β*-cyclodextrin, HP-*β*-cyclodextrin, and HP-*γ*-cyclodextrin (repeated three times).

When extra ketoconazole was added to the solutions of *β*-cyclodextrin, *γ*-cyclodextrin, randomly methylated *β*-cyclodextrin, HP-*β*-cyclodextrin, and HP-*γ*-cyclodextrin in water with different concentrations, after shaking at room temperature for 36 h, the water solubility of ketoconazole in each reaction was determined by HPLC to obtain their phase solubility curves shown in Figure 2. Randomly methylated *β*-cyclodextrin showed the best solubilization effect, and the phase solubility curve can be classified as A_L_ type based on the Higuchi-Connors equation [34].

It was reported that the solubility enhancement by preparing ketoconazole/*β*-cyclodextrin complexes depends largely on the type of preparation methods and conditions [10]. Therefore, the complexations of randomly methylated *β*-cyclodextrin with ketoconazole by the aqueous solution method, ultrasonic method, microwave reactor, autoclave reactor, and supercritical CO_2_ assistance were tried, and the aqueous solution method was found to give the best water solubility enhancement.

The inclusion effect of randomly methylated *β*-cyclodextrin with ketoconazole is commonly affected by the ratio of ketoconazole to randomly methylated *β*-cyclodextrin, reaction temperature, stirring speed, and reaction time. Through single factor and orthogonal strategies (orthogonal design is an experimental design used to test the comparative effectiveness of multiple intervention components), many experimental conditions were designed and performed to prepare the complexes. Based on HPLC analysis of the complex from each condition, the best water solubility was obtained at 290 mg/mL with an 84% inclusion rate and 92.9% inclusion yield, which was 17,000 times better than that of ketoconazole alone and is the best result so far. The reaction conditions include a 1:2 ratio of the drug and cyclodextrin, a stirring speed of 500 r/min, a reaction temperature of 50 °C, and a reaction time of 5 h. The sample was then used for inclusion complex confirmation, in vitro, and in vivo pharmacokinetic studies.

### 2.1. Solid-State Studies

The complex was prepared by using the method discussed above, and after lyophilization, the complex was obtained as a solid.

**Figure 3:**
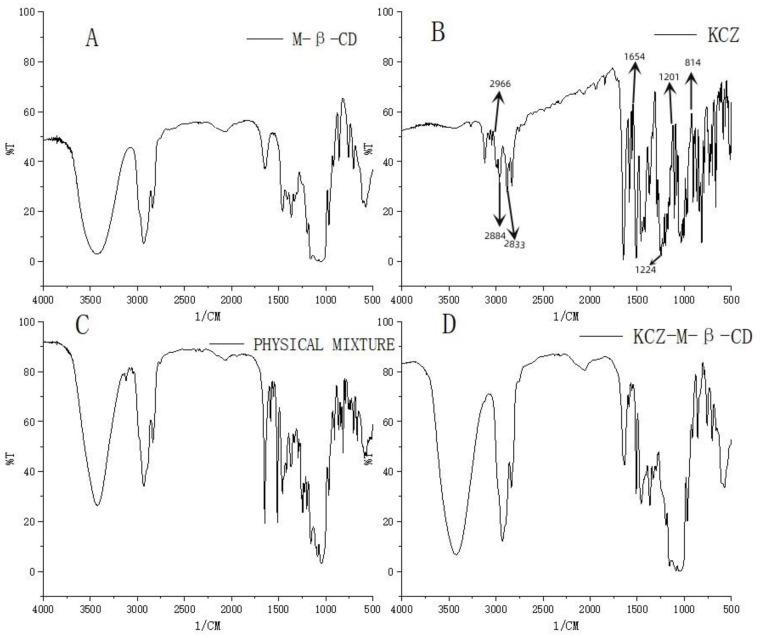
Fourier-transform infrared spectroscopy spectra of randomly methylated *β*-cyclodextrin (**A**), ketoconazole (**B**), the physical mixture of randomly methylated *β*-cyclodextrin and ketoconazole (**C**), and their inclusion complex (**D**).

FTIR spectra were used to confirm the formation of the complex of ketoconazole with randomly methylated *β*-cyclodextrin. As shown in Figure 3, compared with the spectrum of randomly methylated *β*-cyclodextrin (A), the characteristic peaks of ketoconazole appeared at 2966, 2884–2833, 1654, 1224, 1201, and 814 cm^−1^ (B) corresponding to C-H, C=O, C-N, C-O-C, and C-Cl are obviously seen in the physical mixture of ketoconazole and randomly methylated *β*-cyclodextrin (C), and changed (peaks at 1654 cm^−1^, 1224 cm^−1^ and 814 cm^−1^) in the inclusion complex (D), which confirmed the interaction between the ketoconazole and the randomly methylated *β*-cyclodextrin in the complex.

**Figure 4:**
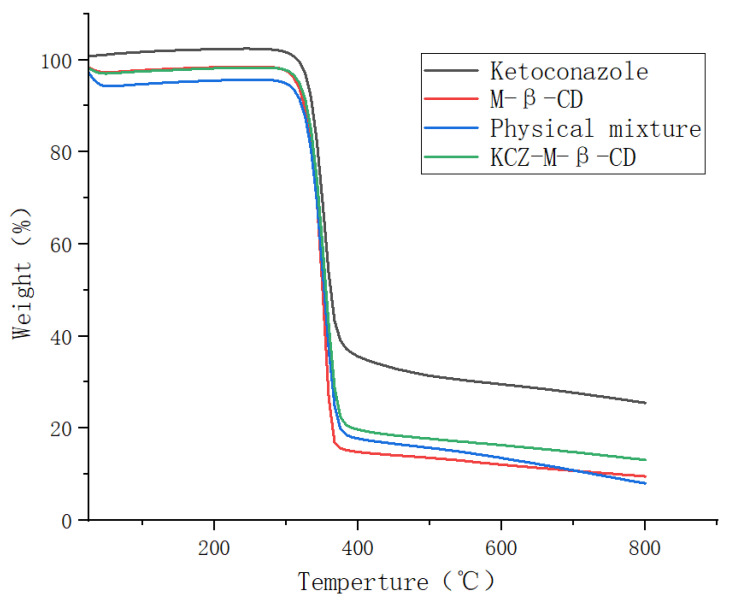
The thermogravimetric analysis of ketoconazole, randomly methylated *β*-cyclodextrin, their physical mixture, and their inclusion complex.

The thermogravimetric analyses were performed on ketoconazole, randomly methylated *β*-cyclodextrin, their physical mixture, and their inclusion complex in the temperature range of 25 °C to 800 °C, as shown in Figure 4. The initial weight losses of four samples before 100 °C were due to water evaporation, and the second mass losses occurring above 300 °C were due to the degradation or deposition of ketoconazole and randomly methylated *β*-cyclodextrin. The formation of the inclusion complex increased the thermal stability of randomly methylated *β*-cyclodextrin and decreased the thermal stability of ketoconazole.

**Figure 5:**
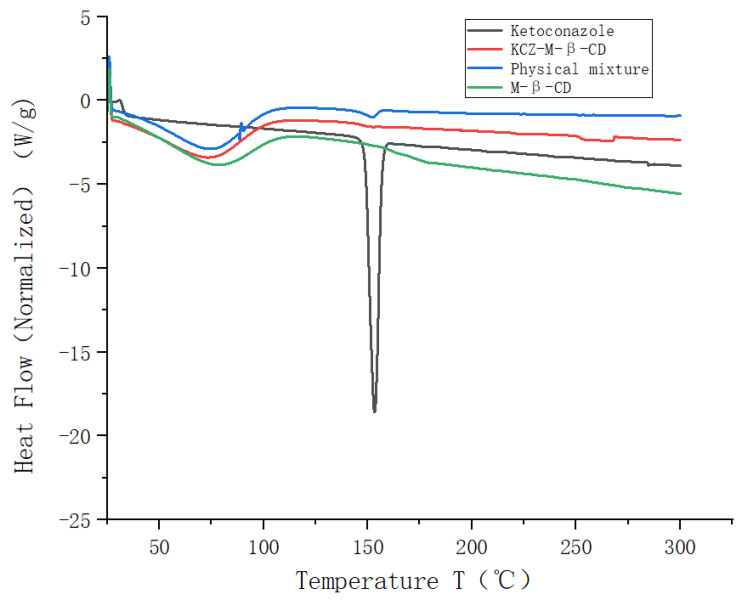
DSC spectra of ketoconazole, randomly methylated *β*-cyclodextrin, their physical mixture, and inclusion complex.

The DSC thermogram obtained for pure ketoconazole exhibited a distinct peak at 151 °C (Figure 5) due to the melting of the ketoconazole. Broad peaks were observed in the thermograms of randomly methylated *β*-cyclodextrin, the physical mixture, and the inclusion complex between 50 °C and 100 °C, assigned to the desolvation of water molecules present in the cavity of randomly methylated *β*-cyclodextrin. The melting endotherm of ketoconazole was seen at 151 °C for the physical mixture. The complete disappearance of the exothermic transition of ketoconazole at 151 °C was found in the thermogram of the inclusion complex.

**Figure 6:**
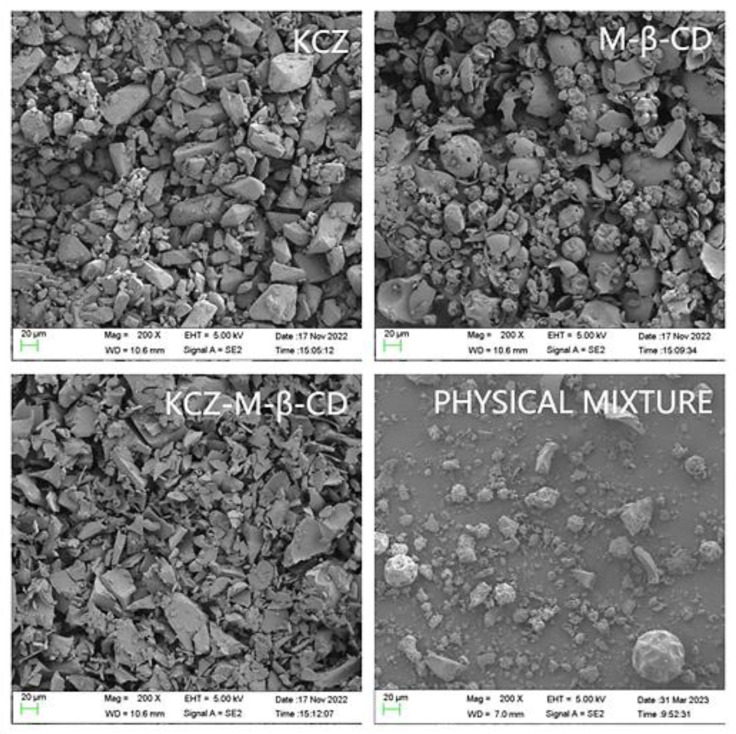
The scanning electron microscope images of ketoconazole, randomly methylated *β*-cyclodextrin, their physical mixture, and their inclusion complex.

A scanning electron microphotograph was utilized to analyze the morphological characteristics of ketoconazole, randomly methylated *β*-cyclodextrin, their physical mixture, and their inclusion complex (Figure 6). The inclusion complex presented the crystals, which were significantly different from the crystals of ketoconazole and randomly methylated *β*-cyclodextrin. The physical mixture prepared with pure powdered ketoconazole and randomly methylated *β*-cyclodextrin presented the crystals of ketoconazole and randomly methylated *β*-cyclodextrin.

All these changes in the morphology of the solid particles, observed in FTIR, TGA, DSC, and SEM, confirmed the complex formation necessary for increasing solubility.

### 2.2. Liquid-Phase Studies

**Figure 7:**
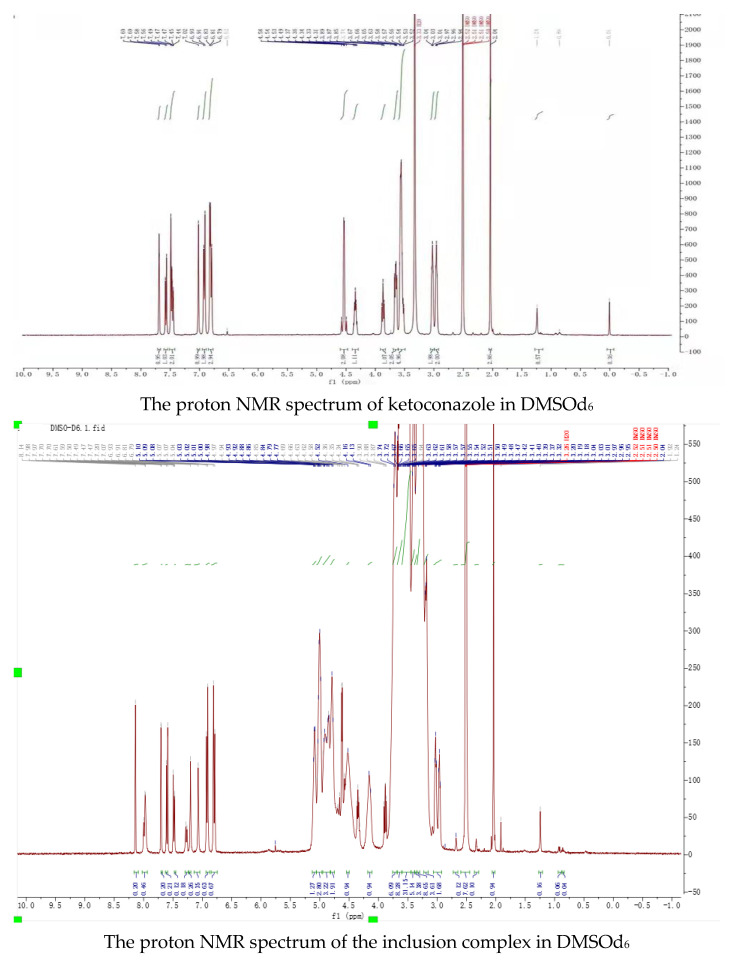

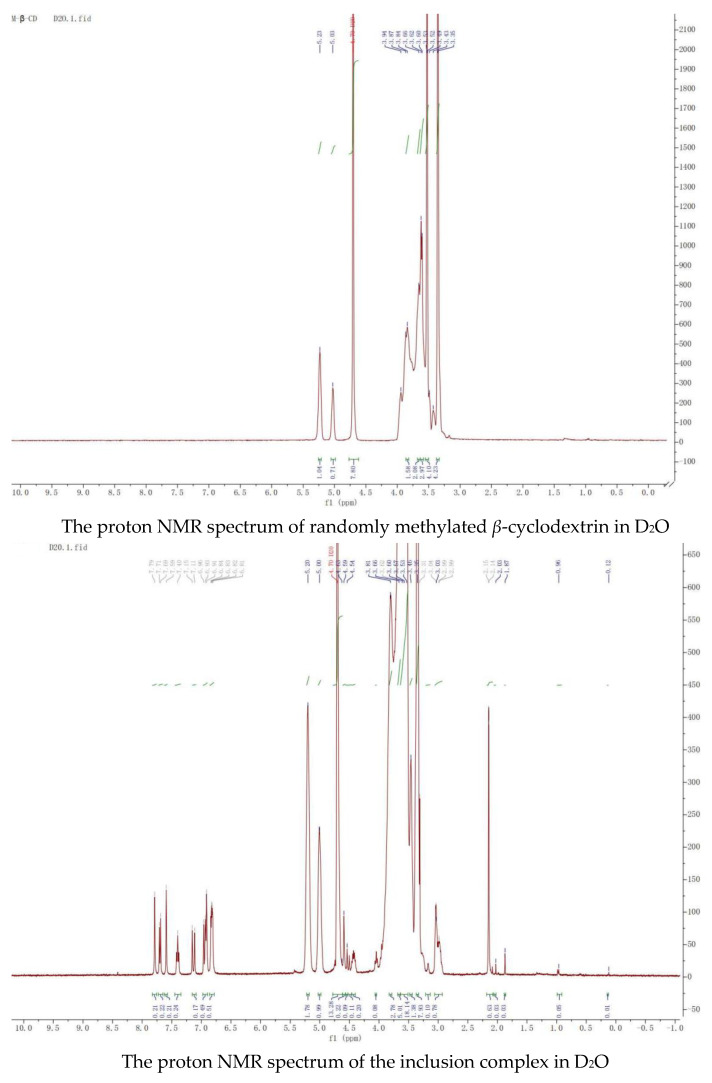

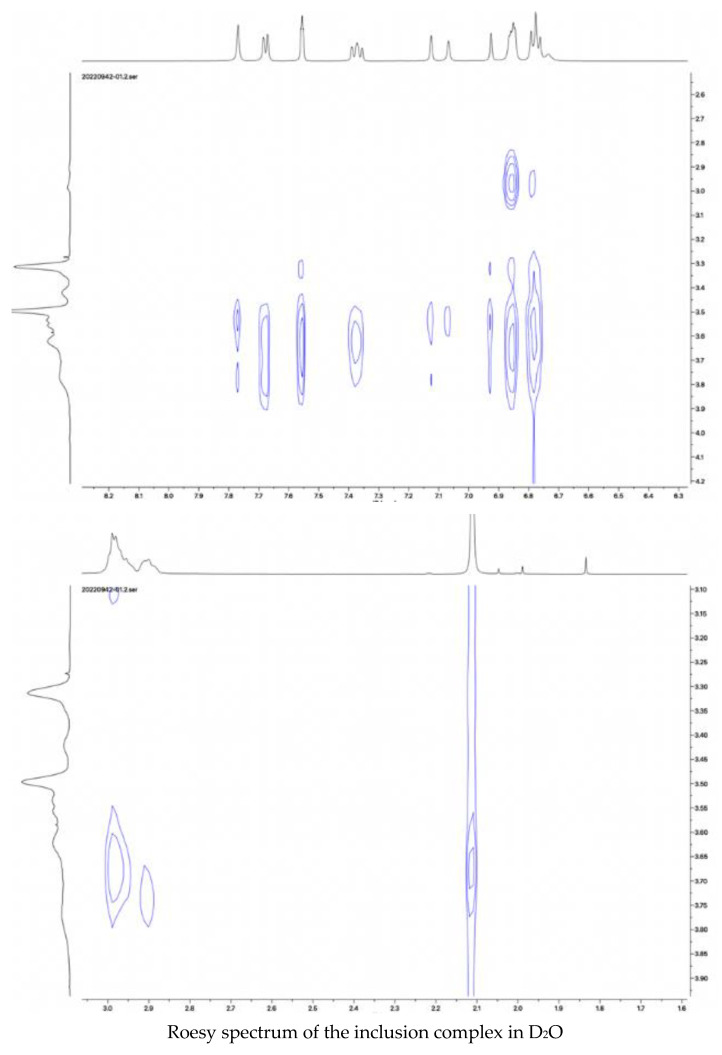
The NMR spectra of randomly methylated *β*-cyclodextrin and its inclusion complex with ketoconazole in D_2_O, the NMR spectra of ketoconazole and its inclusion complex with randomly methylated *β*-cyclodextrin in DMSOd_6_, and the Roesy spectrum of the inclusion complex in D_2_O.

Nuclear magnetic resonance, the most effective method, was used to study the space conformation of the inclusion complex of ketoconazole with randomly methylated *β*-cyclodextrin. The proton NMR spectra of randomly methylated *β*-cyclodextrin and its complex with ketoconazole in D_2_O, the proton NMR spectra of ketoconazole and its complex with randomly methylated *β*-cyclodextrin in DMSOd_6_, and the Roesy spectrum of the inclusion complex in D_2_O were recorded and shown in Figure 7.

Peaks in the proton NMR spectrum of ketoconazole in DMSOd_6_ were assigned as: 7.69 (1H, s), 7.57 (1H, d, *J* = 8.0 Hz), 7.49 (1H, s), 7.46 (1H, d, *J* = 8.0 Hz), 7.02 (1H, s), 6.92 (2H, d, *J* = 8.0 Hz), 6.83 (1H, s), 6.80 (2H, d, *J* = 8.0 Hz), 4.58 (2H, d), 4.34 (1H, t), 3.87 (1H, t), 3.65 (2H, dt), 3.54 (5H, m), 3.03 (2H, t), 2.96 (2H, t), 2.01 (3H, s); the peaks from ketoconazole in the inclusion complex in the proton NMR spectrum in DMSOd_6_ were assigned as: 8.14 (1H, s), 7.70 (1H, s), 7.60 (1H, d, *J* = 8.0 Hz), 7.49 (1H, d, *J* = 8.0 Hz), 7.20 (1H, s), 7.07 (1H, s), 6.92 (2H, d, *J* = 8.0 Hz), 6.80 (2H, d, *J* = 8.0 Hz), 4.66 (2H, d), 4.36 (1H, t), 3.88 (1H, t), 3.04 (2H, t), 2.97 (2H, t), 2.04 (3H, s). Through complexation, the peaks from the dichlorophenyl group were moved from 7.69 ppm, 7.59 ppm, and 7.46 ppm to 8.14 ppm, 7.60 ppm, and 7.49 ppm; the peaks from piperazine groups were moved from 3.03 ppm, 2.96 ppm to 3.04 ppm and 2.97 ppm; the peak at 2.01 ppm from the acetyl group was moved to 2.04 ppm, indicating that these groups were in the cavities of randomly methylated *β*-cyclodextrin.

Peaks from ketoconazole in the inclusion complex in the proton NMR spectrum in D_2_O were assigned as: 7.79 (1H, s), 7.70 (1H, d, *J* = 8.0 Hz), 7.59 (1H, s), 7.40 (1H, t, *J* = 4.0), 7.13 (1H, d, *J* = 16.0 Hz), 6.95 (1H, d, *J* = 16.0 Hz), 6.91 (1H, s), and 6.83 (3H, m). The Roesy spectrum of the complex in D_2_O showed interactions between the protons at 7.70 ppm, 7.59 ppm, and 7.40 ppm from the dichlorophenyl group, at 6.92 ppm and 6.82 ppm from the phenolic structure, at 3.30 ppm from the piperazine structure, at 2.15 ppm from the acetyl group, and the protons at 3.45–3.80 ppm from the cyclodextrin structure, confirming the formation of the inclusion complex. Based on the ratio of the protons from ketoconazole to the protons at C-1 in randomly methylated *β*-cyclodextrin, it was assumed that the inclusion complex contained two molecules of randomly methylated *β*-cyclodextrin and one molecular of ketoconazole.

It is very interesting that even though more than thirty research papers have been published on increasing the water solubility of ketoconazole by complexing it with cyclodextrins, the results are still not significant. In our case, the complex shown in Figure 1 prepared by using the methods reported in the literature could increase the water solubility of ketoconazole by as much as 17,000 times. The increase in water solubility is dependent on the formation of the complexes with cyclodextrins. Many factors, such as cyclodextrin type, complexation methods, reaction time, stirring speed, reaction temperature, concentrations, and the ratio between ketoconazole and cyclodextrins, can affect the formation of the complexes.

### 2.3. The In Vitro PK Studies

**Figure 8:**
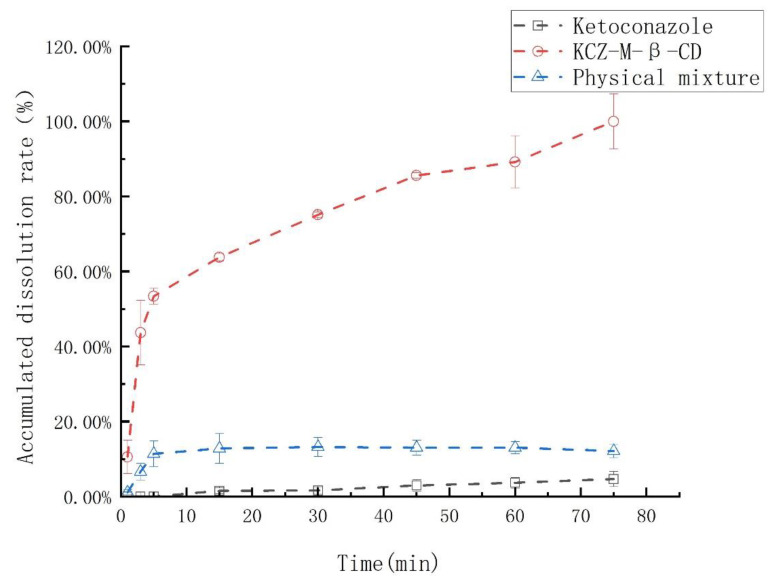
The dissolution rate curves of ketoconazole, the physical mixture of ketoconazole and randomly methylated *β*-cyclodextrin, and their inclusion complex (repeated three times).

The dissolution rates of ketoconazole, the physical mixture of ketoconazole and randomly methylated *β*-cyclodextrin, and their inclusion complex were examined and summarized in Figure 8. The ketoconazole in the inclusion complex was 100% released in 70 min; however, under the same conditions, ketoconazole in the physical mixture was released less than 10%, and ketoconazole was only released less than 1%.

After complexation with randomly methylated *β*-cyclodextrin, the odor and irritation of ketoconazole disappeared since the ketoconazole was ensconced in the randomly methylated *β*-cyclodextrin cylinder, and the contact between randomly methylated *β*-cyclodextrin and taste sensors was inhibited.

### 2.4. The In Vivo PK Studies

**Figure 9:**
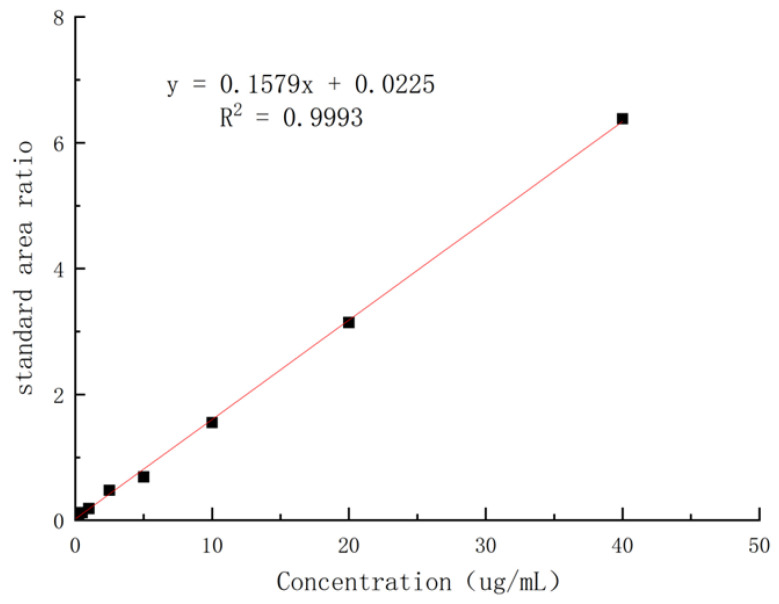
The standard curve of ketoconazole in blank dog plasma.

The solutions of ketoconazole in the blank dog plasma with different concentrations were extracted by using the standard method to provide the samples for HPLC analysis, and it was found that, in the range of 0.05–40 µg/mL, the concentrations of ketoconazole in plasma and the absorption areas in HPLC showed a linear relationship. The regression equation was obtained as Y = 0.1579x + 0.0225 (coefficient of determination: R^2^ = 0.9993, Y: absorption peak area, X: concentration of ketoconazole) with 0.05 µg/mL of LLOD (S/N ≥ 3) and 0.15 µg/mL of LLOQ (Figure 9).

**Figure 10:**
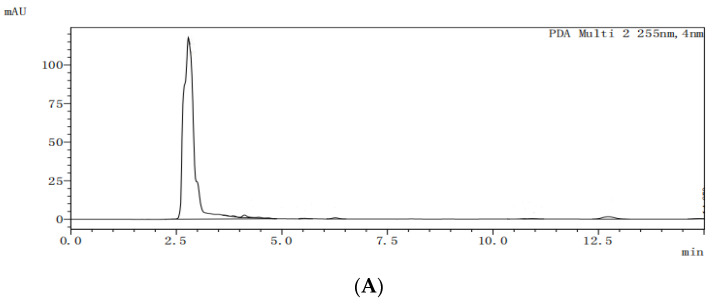

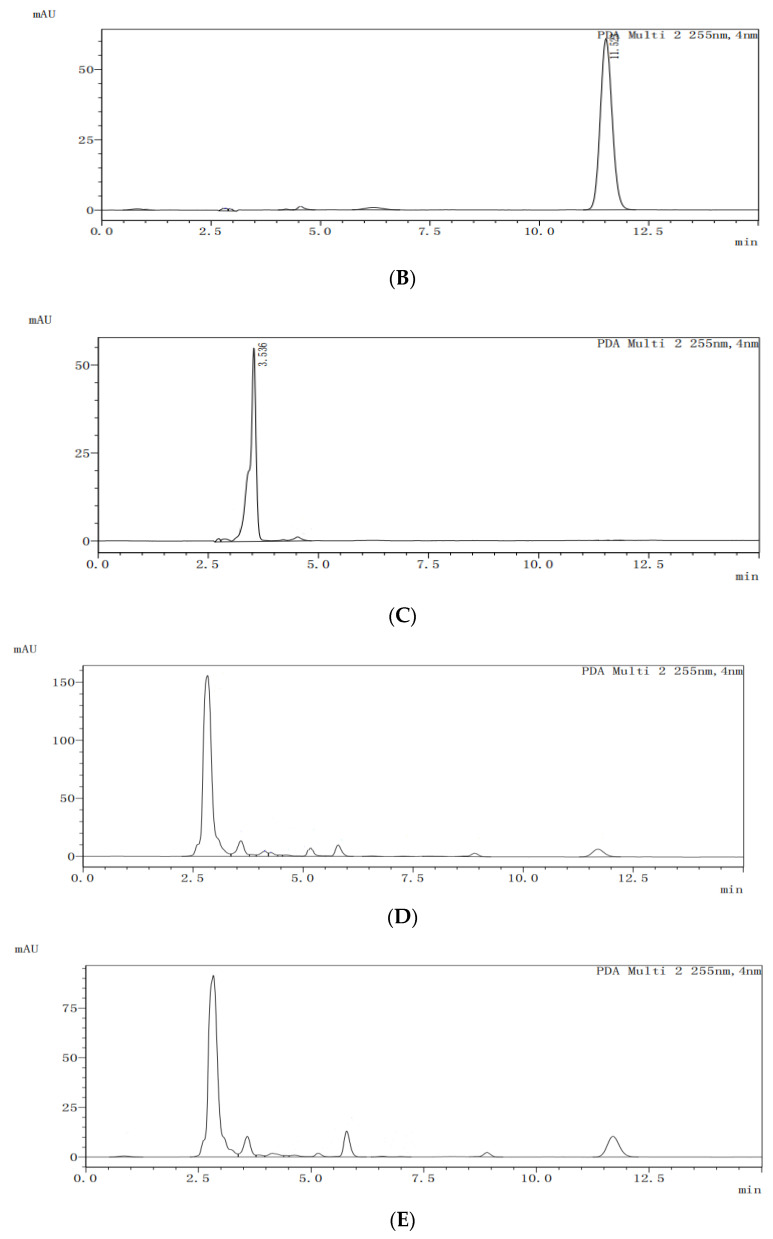
HPLC analysis spectra of blank dog plasma (**A**), ketoconazole (**B**), internal standard (**C**), the internal standard in the plasma from the dogs dosed with ketoconazole (**D**), and the internal standard in the plasma from the dogs dosed with ketoconazole inclusion complex (**E**).

The HPLC analysis spectra of the blank dog plasma (A), ketoconazole (B), the internal standard letrozole (C), the internal standard in the plasma from the dogs dosed with ketoconazole (D), and the internal standard in the plasma from the dogs dosed with ketoconazole complex (E) are shown in Figure 10. Under the selected HPLC conditions, the blank dog plasma did not interfere with the detections of ketoconazole and the internal standard.

The dogs were dosed orally (10 mg/kg) with ketoconazole or its inclusion complex; the blood samples were collected at 0.00 h, 0.25 h, 0.5 h, 0.75 h, 1 h, 2 h, 4 h, 6 h, 8 h, 12 h, 24 h, 48 h, and 72 h, and extracted and analyzed by HPLC.

The recovery rate, intra-assay coefficient of variation, and inter-assay coefficient of variation of ketoconazole in blank dog plasma with concentrations of 10 μg/mL, 20 μg/m, and 50 μg/mL were determined as 95.7 ± 0.53% to 103.4 ± 0.41%, 3.4 ± 0.38% to 5.6 ± 0.52%, and 4.0 ± 0.41% to 5.5 ± 0.53%, respectively.

**Figure 11:**
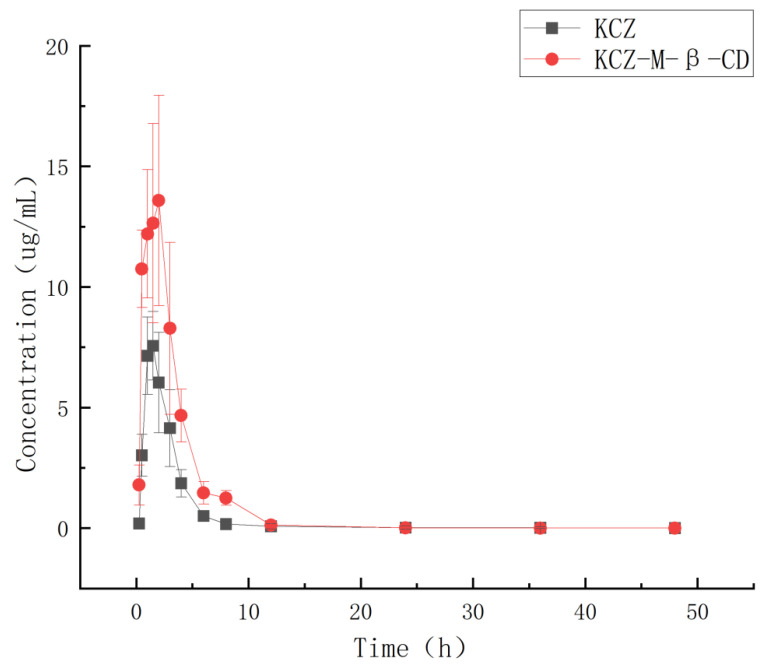
Curves of time-concentration of ketoconazole in plasma from dogs (six dogs for each group) orally administered with ketoconazole or its inclusion complex with randomly methylated *β*-cyclodextrin.

Based on the HPLC analysis data and the regression equation, the curves of time– concentration of ketoconazole in plasma were obtained and shown in Figure 11. The pharmacokinetic parameters (mean ± SD) were obtained based on a non-compartmental model by using Phoenix WinNonlin software (version 8.3) and summarized in Table 1.

After oral administration, the *C*_max_ and *T*_max_ of ketoconazole in dog plasma were changed from 7.56 μg/mL and 1.75 h in the ketoconazole group to 13.58 μg/mL and 1.10 h in the inclusion complex group, and the AUC_0~72_ was changed from 22.69 μgh/mL in the ketoconazole group to 50.19 μgh/mL in the complex group.

Through complexation, the water solubility of ketoconazole was increased 17,000 times compared with that of ketoconazole alone, the *C*_max_ of ketoconazole was increased 1.79 times, the AUC_0~72_ was increased 2.21 times, the *T*_max_ was shortened, the *t*_1/2_ was changed from 1.63 h to 1.98 h, and CL was changed from 1.54 to 0.70, which indicated that less drug is needed for the same therapeutic effect by using inclusion complex, and the ketoconazole inclusion complex produced can be used as a more effective potential new anti-fungal drug.

## 3. Materials and Methods

### 3.1. Regents and Instruments

Ketoconazole (98%) was obtained from Shanghai Macklin Biochemical Co., Ltd., Shanghai, China, *γ*-cyclodextrin, HP-*γ*-cyclodextrin (DS: 4–6), and randomly methylated *β*-cyclodextrin (DS: 11–13) were purchased from Shanghai Macklin Biochemical Co., Ltd., Shanghai, China; *β*-cyclodextrin and HP-*β*-cyclodextrin (average Mw: 1460) were purchased from Shanghai Aladdin Biochemical Technology Co., Ltd., Shanghai, China.

### 3.2. Analytic Methods

UV maximum absorption determination: a solution of ketoconazole in methanol with a concentration of 0.1 mg/mL was vortexed, filtered through a membrane, and scanned by an ultraviolet-visible spectrophotometer (J51903001 from Shanghai Jinghua Technology Instrument Co., Ltd., Shanghai, China) to obtain the UV maximum absorption.

HPLC standard curve establishment: solutions of ketoconazole in methanol with concentrations of 0.06 mg/mL, 0.09 mg/mL, 0.12 mg/mL, 0.15 mg/mL, 0.18 mg/mL, and 0.21 mg/mL were filtered and analyzed by HPLC (LC-15C high performance liquid chromatograph from Shimadzu Enterprise Management China Co., Ltd., Shenzhen, China) with a UV detector (254 nm) on the C-18 column (Inertsil ODS-3 C18: 250 mm × 4.6 mm) to establish the regression equation and the standard curve based on the relationship between the concentrations of ketoconazole and the peak areas in HPLC.

Phase solubility study: 10 mL of solutions of *β*-cyclodextrin, *γ*-cyclodextrin, randomly methylated *β*-cyclodextrin, HP-*β*-cyclodextrin and HP-*γ*-cyclodextrin with concentrations of 5 mmol/L, 10 mmol/L, 15 mmol/L, 20 mmol/L, 25 mmol/L and 30 mmol/L in water were added ketoconazole (10 mg) individually and shaken for 36 h. Each reaction was filtrated to remove the uncomplexed un-soluble ketoconazole and larger-size un-soluble cyclodextrin inclusion complexes and analyzed by HPLC. Each experiment was repeated three times.

### 3.3. Preparation of the Complexes and the Physical Mixture

Aqueous solution method: the solution of randomly methylated *β*-cyclodextrin (1300 mg) in water (15 mL) was added to a solution of ketoconazole (270 mg) in ethanol (10 mL) gradually. After stirring on the magnetic stirrer (RCT digital from IKA) for 5 h at 50 °C, the mixture was kept in a refrigerator at −20 °C overnight, evaporated, supplemented with water (10 mL), filtrated, and freeze-dried to give the product.

Ultrasound method: a solution of randomly methylated *β*-cyclodextrin (1300 mg) in 15 mL of water was added to the solution of ketoconazole (270 mg) in 10 mL of ethanol slowly, ultrasonicated on an ultrasonic reactor (JY92-IIN from Ningbo Xinzhi Biotechnology Co., Ltd., Ningbo, China) at 50 °C with 60%W for 40 min, cooled in a refrigerator at −20 °C overnight, evaporated, supplemented with water (10 mL), filtrated, and lyophilized to obtain the product.

Microwave method: the solution of randomly methylated *β*-cyclodextrin (1300 mg) in water (15 mL) was added dropwise to the solution of ketoconazole (270 mg) in ethanol (10 mL), microwaved on the microcomputer microwave chemical reactor (WBFY-201 from Gongyi Yuhua Instrument Co., Ltd., Gongyi City, China) with stirring (60 r/min) for 30 min, and the reaction was stopped for one min every 30 s. After evaporation, the mixture was supplemented with water (10 mL), filtrated, and lyophilized to generate the product.

Using a hydrothermal reactor: a solution of ketoconazole (270 mg) and randomly methylated *β*-cyclodextrin (1300 mg) in a mixture of ethanol and water (20 mL, *v*:*v* = 2:1) in a hydrothermal reaction kettle (high temperature and pressure reaction kettle BZ-100ML/SC-L from Shanghai Baikal Technology Group Co., Ltd., Shanghai, China) was stirred (1200 r/min) at 120 °C for 10 h. After cooling to room temperature, the mixture was kept in a refrigerator at −20 °C overnight, evaporated, supplemented with water (10 mL), filtrated, and freeze-dried to provide the product.

Using supercritical CO_2_: the solution of randomly methylated *β*-cyclodextrin (1300 mg) in 15 mL of water was added a solution of ketoconazole (270 mg) in 10 mL of ethanol in a reaction kettle (Supercritical carbon dioxide reactor BZ-100ML/S0-L from Baikal Shanghai Intelligent Technology Co., Ltd., Shanghai, China) dropwise, pumped the liquid CO_2_, and stirred at 60 °C for 10 h at a pressure of 8 Mpa. After the pressure was released slowly, the mixture was kept in a refrigerator at −20 °C overnight, evaporated, supplemented with water (10 mL), filtrated, and freeze-dried to give the product.

The physical mixture: the grounded ketoconazole and randomly methylated *β*-cyclodextrin (1:2 in molar ratio) were mixed and passed through an 80-mesh sieve to give the physical mixture.

### 3.4. Experiments through Single Factor and Orthogonal Strategy Strategies

The reaction concentration, the ratio of ketoconazole to randomly methylated β-cyclodextrin, the stirring speed, and the reaction time were fixed; the reaction temperature was changed, and the water solubility was checked for each condition. A reaction temperature of 50 °C was selected.
**Reaction Temperature (°C)****Solubility (mg/mL)**301085028170147

The reaction concentration, the ratio of ketoconazole and randomly methylated *β*-cyclodextrin, the stirring speed, and the reaction temperature were fixed; the reaction time was changed, and the solubility was checked for each condition. A reaction time of 5 h was selected.
**Reaction Time (h)****Solubility (mg/mL)**11375280819212181

The reaction concentration, the ratio of ketoconazole and randomly methylated *β*-cyclodextrin, the reaction temperature, and the reaction time were fixed; the stirring speed was changed, and the solubility was checked for each condition, and a stirring speed of 500 r/min was selected.
**Stirring Speed (r/min)****Solubility (mg/mL)**10078500290100053

The reaction concentration, the reaction temperature, the reaction time, and the stirring speed were fixed; the ratio of ketoconazole and randomly methylated *β*-cyclodextrin was changed and the solubility was checked for each condition. A ratio of 1:2 was selected.
**Ratio****Solubility (mg/mL)**1:11061:22271:3100

The experimental conditions and solubilities through orthogonal strategy study were summarized as follows:

**Stirring sPeed****Reaction Temperature****Reaction Time****Ratio****Solubility (mg/mL)**12005011:111722006051:318032007032:114043505032:17253506051:114663507011:311875005011:229085006032:110495007051:1118

### 3.5. Fourier-Transform Infrared Spectroscopy

Potassium bromide was ground, pressed into tablets as a transparent flake, and scanned by infrared spectroscopy with absorption as the background. Potassium bromide was ground with ketoconazole, randomly methylated *β*-cyclodextrin, their physical mixture, and their inclusion mixture, and subsequently pressed into flakes and scanned by infrared spectroscopy (Fourier Transform Infrared Spectrometer IR Tracer 100 from Shimadzu, Kyoto, Japan) from 500 cm^−1^ to 4000 cm^−1^.

### 3.6. Thermogravimetric Analysis

Around 5 mg of ketoconazole, randomly methylated *β*-cyclodextrin, their physical mixture (ratio: 1:2) and inclusion complex were weighed, ground, passed through an 80-mesh sieve, and analyzed on the thermogravimetric analyzer (Thermogravimetric analyzer TGA2 from Mettler Toledo, Columbus, OH, USA) under nitrogen in the range 25–800 °C at a heating rate of 10 °C/min with nitrogen purge at 50 mL/min.

### 3.7. Differential Scanning Calorimetry Analysis

Around 5 mg of ketoconazole, randomly methylated *β*-cyclodextrin, their physical mixture (ratio: 1:2), and their inclusion complex were weighed, ground, passed through an 80-mesh sieve, and analyzed in a 40 µL standard aluminum crucible on the DSC3 instrument (the differential scanning calorimeter DSC3 from Mettler Toledo, Columbus, OH, USA) under nitrogen purge at 50 mL/min and a heating rate of 10 °C/min in the temperature interval of 25–300 °C.

### 3.8. Scanning Electron Microscopy

The samples of ketoconazole, randomly methylated *β*-cyclodextrin, the physical mixture, and the inclusion complex were placed on the sample stage, gold-plated, scanned, and pictured under 5 kV of the accelerating voltage, respectively, on the SEM imagine instrument (Merlin from German Zeiss Company, Oberkochen, Germany).

### 3.9. NMR Study

The proton NMR spectra of the ketoconazole inclusion complex in D_2_O, randomly methylated *β*-cyclodextrin in D_2_O, ketoconazole in DMSOd_6_, and its inclusion complex in DMSOd_6_ were recorded in the Bruker 400 NMR spectrometer (from Bruker, Karlsruhe, Germany), and the Roesy spectrum of the ketoconazole inclusion complex in D_2_O was recorded in the Bruker 600 NMR spectrometer (from Bruker, Karlsruhe, Germany).

### 3.10. Determination of Water Solubility of Ketoconazole in Complex

The saturated aqueous solution of the inclusion complex of ketoconazole in water was prepared by adding the complex to 1 mL of water, and after shaking for 36 h and filtration to remove non-soluble materials, the solution was analyzed by HPLC to obtain the water solubility of ketoconazole in the complex.

### 3.11. Determination of the Inclusion Rate and Inclusion Yield

The following formulae were used to calculate the inclusion rate and yield of ketoconazole in the inclusion complex:

Inclusion yield (%) = [inclusion complex (mg)/ketoconazole (mg) + cyclodextrin (mg)] × 100%

Inclusion ratio (%) = [ketoconazole in inclusion complex (mg)/ketoconazole (mg)] × 100%

### 3.12. In Vitro Dissolution Rate

The paddle method, based on The Chinese Veterinary Pharmacopoeia 2010 edition, was used to test the dissolution rate. The samples of ketoconazole (100 mg), the physical mixture (581 mg containing 100 mg of ketoconazole), and the inclusion complex (containing 100 mg of ketoconazole) in three release cups (10 mL) in three containers with 900 mL of degassed water as the dissolution medium were stirred on the dissolution tester RC-6 (from Tianjin Xintianguang Analytical Instrument Technology Co., Ltd., Tianjin, China) at 100 r/min and 37 °C (±0.5). One mL of medium was taken from each container at 1 min, 3 min, 5 min, 15 min, 30 min, 45 min, 60 min, and 75 min, filtered with a 0.22 μm microporous membrane for 30 s for HPLC analysis, and 1 mL of dissolution medium was then added to the container at the same temperature and same time. Based on the HPLC analysis data and the time the sample was taken, the in vitro dissolution rate curve was obtained.

### 3.13. In Vivo Pharmacokinetic Study

Healthy adult dogs (12) weighing around 5 ± 0.1 kg, half male and half female, were randomly divided into two groups (six for each): the drug group and the complex group. They were numbered and fed for one week in a warm and ventilated environment. Before drug administration, the dogs were fasted for one day, and after the drug administration, the dogs were fed low-fat dog food and normal drinking until the sample collections were finished.

After dogs were given a single dose of ketoconazole (10 mg/kg) or complex (effective content of ketoconazole: 10 mg/kg) orally by mixing the drug or complex with water, the blood samples (2 mL) were collected through the anterior vena cava at 0.00 h, 0.25 h, 0.5 h, 0.75 h, 1 h, 2 h, 4 h, 6 h, 8 h, 12 h, 24 h, 48 h, and 72 h by using negative pressure disposable blood collection tubes filled with heparin sodium, transferred to centrifuge tubes for centrifugation at 13,000 r/min for 10 min. The collected supernatant plasmas were then numbered and stored at −20 °C in the refrigerator.

After the plasma sample was thawed to room temperature, 0.2 mL of it was pipetted into a centrifuge tube (2 mL), and the internal standard of 10 μL (letrozole, 500 μg/mL in acetonitrile) was added. After adding 1 mL of acetonitrile, the mixture was vortexed for 2 min, centrifuged at 13,000 r/min for 10 min, and transferred into the test tube. The extraction procedure was repeated twice. The resulting solution was transferred to a test tube (10 mL), blown by nitrogen at 50 °C to dryness, added to the mobile phase (0.5 mL), vortexed for 5 min, and filtered through a 0.22 μm microporous membrane to provide the sample for HPLC analysis.

### 3.14. Establishment of the Standard Curve of Ketoconazole in Plasma

A solution (1 mL) of ketoconazole in methanol with a concentration of 1 mg/mL was added to 0.2 mL of the blank plasma and diluted with HPLC mobile phase to prepare a series of plasma solutions with ketoconazole concentrations of 0.5 μg/mL, 1 μg/mL, 5 μg/mL, 10 μg/mL, 20 μg/mL, 50 μg/mL, 80 μg/mL, and 100 μg/mL. Each sample was extracted by using the method described above to obtain samples for HPLC analysis (wavelength: 254 nm; column: Shim-packVP-ODS C18 chromatographic column 250 × 4.6 mm; mobile phase: 25:75 of 0.0036 mol/L potassium dihydrogen phosphate water and methanol; flow rate: 1.0 mL/min; temperature: 35 °C; injection volume: 10 μL). Based on the concentrations and peak areas of the samples in HPLC, the standard curve and equation of ketoconazole in plasma were obtained.

### 3.15. Statistical Analysis

All the experiments were carried out in triplicate; the average value was used for analysis, and the results were expressed as mean ± standard deviation. Every point in the pharmacokinetic curve represents the average of six experiments, and the statistical analysis was performed by one-way analysis of variance followed by a significant difference test using Prism 9 software; *p* ≤ 0.05 means the difference is considered statistically significant.

## 4. Conclusions

Ketoconazole, a powerful antifungal drug, was used to inhibit the production of fungal cell membranes and treat a diversity of deep and superficial tissue fungal infections; however, due to its poor water solubility, its clinical applications were greatly limited. The inclusion complex of ketoconazole with randomly methylated *β*-cyclodextrin was prepared after cyclodextrin selection by using phase solubility studies, complexation methods, and condition selection with single factor and orthogonal strategies, and confirmed by FTIR, TGA, DSC, SEM, and NMR studies. Through complexation, the water solubility of ketoconazole was increased 17,000 times compared with the drug alone, which is so far the best result for ketoconazole’s water solubility study, ketoconazole was 100% released in 75 min in vitro, and in vivo pharmacokinetic study in dogs, the *C*_max_ of ketoconazole was increased 1.79 times, AUC_0~72_ was increased 2.21 times, half life time was changed from 1.68 h to 1.98 h, the clearance rate was changed from 1.54 to 0.70, which indicates that less drug is needed to keep the same therapeutic effect, and this complex can be used as a potential potent new anti-fungal drug, its toxicity study, and in vivo efficacy study will be started in due course.

## Figures and Tables

**Table 1:** Pharmacokinetic parameters (mean ± SD) of ketoconazole and its inclusion complex.

Parameter	Unit	Value	
		KCZ	Complex
*T*_max_	h	1.75 ± 0.25	1.10 ± 0.68
*C*_max_	µg/mL	7.56 ± 2.08	13.58 ± 4.36
AUC_0~72_	µgh/mL	22.69 ± 9.51	50.19 ± 14.98
*t*_1/2_	h	1.63 ± 0.50	1.98 ± 1.03
CL	L/kg/h	1.54 ± 0.44	0.70 ± 0.33

## Data Availability

Data is contained within the article.
